# Perspectives of Volunteer Firefighters during the COVID-19 Pandemic: Stumbling Blocks and Silver Linings

**DOI:** 10.3390/challe13020046

**Published:** 2022-09-09

**Authors:** Alice A. Gaughan, Laura J. Rush, Sarah R. MacEwan, Ashish R. Panchal, Ann Scheck McAlearney

**Affiliations:** 1 The Center for the Advancement of Team Science, Analytics, and Systems Thinking (CATALYST), College of Medicine, The Ohio State University, Columbus, OH 43210, USA; 2 Division of General Internal Medicine, College of Medicine, The Ohio State University, Columbus, OH 43210, USA; 3 Department of Emergency Medicine, The Ohio State University Wexner Medical Center, Columbus, OH 43210, USA; 4 Department of Family and Community Medicine, College of Medicine, The Ohio State University, Columbus, OH 43210, USA; 5 Department of Biomedical Informatics, College of Medicine, The Ohio State University, Columbus, OH 43210, USA

**Keywords:** COVID-19, vaccination, vaccine hesitancy, emergency medical service, mental health

## Abstract

The COVID-19 pandemic has profoundly affected the lives of almost every individual in every nation, with numbers of infections continuing to grow. Across these nations, first responders are essential in their roles addressing emergencies, despite their risk of exposure to COVID-19 in the course of their work. We sought to understand the impacts of the COVID-19 pandemic on the lives of volunteer firefighters in the United States, an understudied group of these first responders. Interviews were conducted with volunteer firefighters between September and November 2021. Interviews were analyzed using deductive dominant thematic analysis. Thirty-three firefighters were interviewed who had an average of 22 years of service and a mean age of 52 years. Interviewees described pandemic-related challenges including the fear of COVID exposure and frustrations with work and personal relationships. They also identified unexpected work-related benefits including a deepened commitment to serve and improvements to training and safety. Further, some volunteers noted personal benefits such as developing stronger connections with others, having a new outlook on life, and observing goodwill. Our findings provide insight into the multifaceted and complex impact of the COVID-19 pandemic on volunteer firefighters.

## 1. Introduction

The COVID-19 pandemic has exacted a massive toll on the health and well-being of the United States (US) population. To date, the negative impacts of COVID-19 have been examined in racial and ethnic minority groups [1,2], US immigrants [3], gender minority groups [4], pregnant women [5], and those marginalized by the digital divide [6]. Additionally, among specific segments of the US healthcare workforce, increased risk of exposure to COVID-19 has been demonstrated in many types of essential workers [7], and psychological stress has been documented in frontline hospital workers [8], physicians [9,10], and pharmacists [11]. These studies and others show the widespread, pervasive consequences of the pandemic and indicate that society will feel repercussions for years to come.

First responders play an essential role in society by responding to fire, medical, hazardous material, or other similar emergencies. Less is known about the impact of COVID-19 on first responders despite their risk of exposure performing their work duties. One recent study estimated that more than half of the workers in protective services, which includes firefighters, were exposed to COVID-19 at least once a month, and nearly one-third of these workers would be exposed at least once a week [7]. Studies have shown that many first responders experience stress and anxiety about potential exposure and putting their families at risk [12]. Furthermore, researchers have compared the mental health issues resulting from the 11 September 2001 attacks to what first responders are experiencing with the COVID-19 pandemic [13].

COVID-19 stress and anxiety experienced by first responders may not be remedied by the availability of vaccines against COVID-19 if the vaccines are not widely accepted among these groups. Prior to vaccine availability, a survey of firefighters and emergency medical services (EMS) personnel showed that fewer than half of respondents (48.2%) indicated high acceptability of a COVID-19 vaccine if it were to become available [14]. More recently, a survey conducted after emergency use authorization of COVID-19 vaccines found that vaccine hesitancy was still high (30%) among EMS personnel [15,16].

Of note, there are approximately 745,000 volunteer firefighters in the US [17]. In the US, volunteerism is critical for the public medical safety net with most volunteer firefighters working in rural settings or serving areas with fewer than 25,000 people [17–19]. This group is predominantly male (89%), and nearly half are 40 years of age or older. In practice, volunteer firefighters may handle exclusively fire calls or respond to both fire and medical calls, and both may result in physical and mental stress. Firefighting can also increase one’s risk for cardiovascular and pulmonary disease, which are both known risk factors for COVID-19 illness [20]. Furthermore, many of these individuals also work in paid positions in addition to serving as volunteers, potentially contributing to their exposure risks and stress.

The stress of being on the front lines delivering emergency services combined with the potential for exposure to COVID-19 in chaotic situations may place volunteer firefighters at high risk for adverse physical and psychological effects resulting from the COVID-19 pandemic. We designed this study to improve our understanding of the impacts of this pandemic on volunteer firefighters in relation to both their volunteer service roles and as members of their local communities.

## 2. Materials and Methods

### 2.1. Study Design and Setting

We conducted a qualitative study comprised of interviews with members of the National Volunteer Fire Council (NVFC), a nonprofit membership association for volunteer fire, EMS, and rescue services. Interviews were held between September and November 2021 and aimed to understand how NVFC members were both professionally and personally impacted by the COVID-19 pandemic.

### 2.2. Study Participants, Data Collection, and Interview Procedures

NVFC members, including volunteer firefighters and emergency medical technicians (EMTs), were recruited to participate in study interviews with the help of NVFC leadership. All NVFC members (approximately 12,370) received a recruitment email from their organization with information about the study and how to participate. Interested members contacted the study team to participate in the study and were scheduled for interviews in the order they expressed interest. Although many NVFC members expressed interest in participation, interviews ceased once we reached saturation in data collection; this gives us confidence about the salience of the results we present [21].

All interviewees provided informed consent to participate in the study. Interviews were conducted by phone using a semi-structured interview guide that asked questions about the impact of COVID-19 on NFVC members. Interviews lasted approximately 40 min and were audio-recorded, transcribed verbatim, and de-identified.

### 2.3. Data Analysis

Interview transcripts were coded and analyzed using deductive dominant thematic analysis [22–25], allowing for categorization of data based on general themes derived from the interview guides, as well as identification of emergent themes. This approach allowed for a comparison of themes across interviews and enabled us to characterize the impact of the COVID-19 pandemic on volunteer firefighters. We used ATLAS.ti version 9.1.3 (ATLAS.ti Scientific Software Development GmbH, Berlin, Germany) qualitative data analysis software to support our coding and analysis process. Considering NVFC members’ experiences as first responders during the COVID-19 global pandemic, we were interested in perspectives about how COVID-19 has impacted their volunteer work and their personal lives, as we report below.

## 3. Results

### 3.1. Characteristics of Study Participants

We interviewed a total of 33 NVFC members across 24 states. On average, interviewees had volunteered for their local fire services for 22 years and were 52 years of age (range of 27–73 years; SD: 12). Most interviewees were male (73%), nearly half had completed EMS/EMT medical training (45%), and about one-third achieved the rank of lieutenant or higher (33%). Table 1 presents characteristics of interviewees by gender and age.

### 3.2. Work Challenges: Perceived “Stumbling Blocks” of the COVID-19 Pandemic

Data collected from our study interviews revealed that the COVID-19 pandemic posed important challenges for volunteer firefighters in their roles as first responders. Interviewees discussed work challenges including: (1) fear of COVID exposure and transmission during emergency response; (2) frustration with emergency responders not following public health measures; and (3) difficulty fostering camaraderie. We describe these themes in further detail next, and present additional verbatim quotations in Table 2.

First, volunteer firefighters acknowledged fear about COVID-19 exposures during emergency responses. As one EMT highlighted, *“The pandemic has increased stress levels. So now there’s even times when I go on what we call a speed call. In other words, I’m not on duty, but they need somebody to respond. I’ll respond, and I don’t always have all my protective gear with me. So, yesterday I was on a call for a guy laying on the side of the road, and all I had was gloves. Okay so this guy is vomiting. Does he have COVID? I don’t know. Just don’t let them breathe on me”*. This fear of COVID-19 exposure also reportedly discouraged some volunteers from responding to emergency calls. As one firefighter explained, *“Some people will really decrease their [emergency] responses because they just, you know, they may have somebody at home with a weakened immune system or something. So, they’ve decided that they’re not going to participate and risk bringing that back home to their family”*.

Second, volunteer firefighters expressed disappointment with some of their coworkers who did not follow public health measures against COVID-19. As one firefighter reflected about his coworkers refusing to get vaccinated, *“I was shocked. It wasn’t the general public. These are firefighters* … *it’s disappointing. I guess in many ways it’s heartbreaking. You know, it’s, I have the same kind of feelings about people who work in the healthcare field resisting vaccination. I’m like, how can you ethically say that you want to care for people, help people, protect people, and not do something that is so simple to try to keep the health of others in your community safeguarded? It just seems to go against everything that we’re supposed to stand for”*. An EMT firefighter expressed similar frustrations by sharing, *“Not everybody following the guidelines and documents, you know, that’s the hard part, you know, that’s most frustrating”*.

Volunteer firefighters’ responses also suggested that connecting and building trust with coworkers was much more difficult during the COVID-19 pandemic. They commented on how the changes during the pandemic resulted in less camaraderie among the firefighters. As one firefighter reflecting on the changes during the pandemic shared, *“We used to look forward to seeing each other, hanging out after calls, talking, catching up with everybody and now it’s like, go do the call, and let’s get everybody out of here. So, it’s changed the camaraderie a lot”*. Similarly, another firefighter explained *“Well, I think we’ve lost a lot of connection to one another. That [socializing and cooking at the firehouse] is really when, you know, a lot of the camaraderie and the trust building, and the connection happens. Certainly, it happens during training. Certainly, it happens during calls. But it’s all that soft stuff that happens in addition to what you have to do, where you really start to get connection and a feeling of tightness with your department. And that was being impeded for many months”*.

### 3.3. Personal Challenges: Perceived “Stumbling Blocks” of the COVID-19 Pandemic

Volunteer firefighters also described the negative impacts the COVID-19 pandemic was having on their personal lives such as: (1) fracturing of relationships; (2) disruptions to their social support system; (3) chronic stress and negative impacts on mental health; and (4) feeling frustrated that people are not taking the pandemic seriously. We describe these themes in further detail next and present additional verbatim quotations in Table 3.

Interviewees’ responses suggested they noted a fracturing of some relationships that was related to issues surrounding the COVID-19 pandemic. As one firefighter reflected, *“I lost a few friends, because of this. We don’t talk to each other anymore. Their belief system is so opposite of each other that we can’t talk to each other because they become angry. Well, hopefully in a few years they’ll come back. We’ll have a nice beer together and slap each other and say how in the heck did we get this way? That means we were true friends to begin with”*. Another interviewee shared, *“So, we now, unfortunately, know who in our previous group of friends did not want to get a vaccine or had some weird out-of-left-field thoughts. And so, moving forward, that’s just going to be an issue that we’re going to have to encounter. And either find a way to look past that with people, or our social group is just going to become smaller and smaller with people who think more like us”*.

Volunteer firefighters’ responses also suggested negative effects for themselves and others caused by disruptions to their social support system. One firefighter reflected on how a lack of social gatherings impacted her and her spouse, *“Yes, I think that the inability to responsibly be social with a large group of people has impacted us. It was initially very stressful to not be able to be out with a larger group of friends, but we have learned to cope with that enclosed social group and now it’s okay. But at the beginning it was very stressful”*. Another firefighter described additional impacts on the social relationships of children, *“What it’s going to do to our young children that have seen that have been through this… They are stuck at home for school for a whole year or something. How are these kids going to handle being socially out in public?”*.

Further, many volunteer firefighters expressed concern about chronic stress and the negative mental health impacts of the COVID-19 pandemic. One interviewee shared, *“I have a tendency to be a whole lot more angry. Yeah, I mean when I go home and my husband is watching the news, I’m like, ‘Turn that crap off!’ And the anger comes to the surface”*. Another interviewee reflected similarly, *“We’re just over it, ready for it to be done. You can only stay super vigilant for so long”*.

Finally, volunteer firefighters commented they were frustrated by people not taking COVID-19 seriously. They shared their frustrations about people in the community who were against wearing masks and increasing risk to others. One interviewee commented, *“I think there’s a frustration just, you know, just kind of how, as a community, and, including the wider community, how we’re dealing with it, and not dealing with it at times. It’s a little frustrating when some folks are kind of cavalier about it. And I’ve had, you know, I have relatives that have a relative who passed away. I understand it’s their choice, but it is a little frustrating when some of the choices that they make impact others unnecessarily”*. Another interviewee shared similar frustrations in reflecting, *“I’m a little more frustrated that people are still thinking that this is just a light case of the flu, or, you know, that the people who are denying that this is an epidemic, pandemic. I get frustrated with that, you know, they’re not taking steps to protect themselves or others”*.

### 3.4. Work Benefits: Perceived “Silver Linings” of the COVID-19 Pandemic

Despite the devastating impacts of COVID-19 on society, volunteer firefighters also reported finding “silver linings” that they associated with their experiences during the pandemic. Specifically, they mentioned unexpected benefits related to their work including: (1) a reinforcement of commitment to serve as a volunteer firefighter, and (2) an opportunity to improve training and work safety. We describe these themes in further detail next and present additional verbatim quotations in Table 4.

First, responses from volunteer firefighters suggested they experienced a renewed commitment in themselves and others to serve as emergency responders. As one interviewee reflected, *“We are one of the few volunteer departments in the area that actually has a waiting list of people to get on. We just have a lot of new younger guys and girls in the community that just want to step up and serve. And maybe this COVID’s actually helped because they see the need for it”*. Another interviewee shared, *“So, there’s another thing that pops up with COVID, it says, ‘Oh, this is a life event where you should reconsider whether you’re a firefighter.’ The benefit of providing service to the community outweighs the personal risk that I’m going to accept”*.

Interviewees additionally suggested that the COVID-19 pandemic provided a benefit for emergency responders by enabling improvements to volunteer firefighter training and safety. One volunteer firefighter commented, *“I think it’s done a number of things, obviously it’s, you know, changed our work environment. A lot more stuff has gone remote. I think it’s opened our eyes to some things that we were doing in the past that probably weren’t the most efficient ways that we could be doing things. I think we’re foolish not to look at some of the things and say has this, corrected maybe, some things that could have used some correction for a long period of time”*. Another volunteer firefighter shared, *“Firefighters think they’re invincible and firefighters sometimes get a little too relaxed when it comes to taking in all aspects of safety. But, even safety in the medical sense. And I think this has caused us just to kind of reevaluate some of the unnecessary risks that we’ve taken and to be more cognizant of the safety measures that we’ve been taught and take them a little more seriously. Unfortunately, it took some people we know dying in order for that to happen”*.

### 3.5. Personal Benefits: Perceived “Silver Linings” of the COVID-19 Pandemic

Volunteer firefighters also mentioned personal benefits resulting from the pandemic, such as (1) developing stronger connections with others; (2) having a new outlook on life; and (3) observing the best of our society. We describe these themes in further detail next and present additional verbatim quotations in Table 5.

Volunteer firefighters commented about how they perceived a “silver lining” in their ability to develop stronger connections with others during the pandemic. One interviewee shared, *“The social family, the people in your household, relationships have gotten stronger because people are forced to spend more time together”*. Similarly, another interviewee explained, *“So, it’s opened up some beautiful ways of building connections with people, sharing stuff”*.

Interviewees also reported having a new outlook on life in response to the COVID-19 pandemic. Several volunteer firefighters shared decisions they made in their personal lives. For example, one volunteer firefighter said, *“I think it’s turned out to be a very positive year, year and a half, whatever, for us to just kind of relax, enjoy life more. I got a new position at work and have been able to focus more on that. And, we don’t have the stress of going into our jobs. We don’t have to stress about going out on weekends. And, I think it’s just an overall positive. It was hard at first, but we got past that pretty quickly”*. Another volunteer firefighter pointed out, *“I think it’s just made me more appreciative of life, of the people around me and understanding life can be short”*.

Last, interviewees shared that their experience during the pandemic involved seeing some of the best of our society. Many volunteer firefighters felt proud about their role in helping community members during the crises. A fire chief noted, *“A lot of people in this business have done extraordinary things in life or in face of the threats of this pandemic, and much like what happened on 9/11 in our business, they’ve really come through. They’ve shown the best of our society, and I hope that our communities are as proud of their volunteer firefighters as I am, because what they have done has been nothing short of amazing”*. Similarly, a volunteer firefighter EMT explained, *“I took good care of myself to make sure I didn’t get it so I could still help other people”*.

## 4. Discussion

Our study shows the broad impact of COVID-19 on both the professional and personal lives of volunteer firefighters who have been working throughout this pandemic. In spite of ongoing burdens from the pandemic and added danger to themselves and their coworkers from potential occupational exposure to COVID-19, many study participants noted that they had also seen some benefits from this experience. Of note, we did not ask participants directly whether they had observed anything positive during this time frame; rather, they spontaneously provided examples of good things that had occurred because of the emergence of COVID-19. Interestingly, several of the stumbling blocks and silver linings that we identified represented opposing views of similar topics: some relationships were lost while others were strengthened; chronic stress negatively impacted mental health yet brought a new outlook on life; frustration was felt for those not acting responsibly during the pandemic while inspiration was felt for those who stepped up to help in this time of need.

Recently, DePierro, et al. wrote about the mental health consequences such as the post-traumatic stress disorder (PTSD) and depression that first responders and medical personnel developed following the attacks of 11 September 2001 and have suggested that frontline workers and essential personnel may experience similar effects from the COVID-19 pandemic [13]. Our study participants reported feeling chronic stress and perceiving negative impacts of the COVID-19 pandemic on their mental health caused, at least in part, by their volunteer roles. What is less clear, however, is what support is available to them through their volunteer organizations to help them address the unique challenges brought on by the pandemic. National programs are available to support the mental wellbeing of firefighters [26,27]. However, it is important to recognize that access to support, such as to behavioral health care, differs for professional and volunteer firefighters [28], and volunteer organization may not have the resources to provide adequate access. Ensuring resources are available to all firefighters, regardless of their professional or volunteer status, is critical when the toll on mental health can be reflected in serious signs of distress including a high prevalence of suicidal thoughts and behaviors [29].

There is ample evidence suggesting that strong social connections in the firehouse contribute significantly to resilience, work performance [30–32], and better health outcomes [33]. However, some of our study participants expressed concern and dismay that safety precautions (physical distancing, masks, decreased time spent in the company of others) as well as differences in beliefs about COVID-19 in general, and vaccinations specifically, were contributing to a lack of work camaraderie. This is consistent with findings from other research that has shown vaccine hesitancy among firefighters and EMS personnel [14,15,34], which has contributed to a societal chasm between vaccinated and unvaccinated people, including within the emergency responder workforce [15,16,35]. As the pandemic has continued, attention needs to be paid to how this protracted disruption to the social fabric of firehouses and/or individual relationships within those firehouses that has resulted from the COVID-19 pandemic may have negative consequences for sustaining the volunteer firefighter workforce.

The perspectives of our study participants also suggest possibilities for post-traumatic growth, described as positive change experienced due to a traumatic experience [36]. In practice, the continuing perceived threat of COVID-19 and strong social connections have both been shown to increase the possibility of post-traumatic growth [37]. This suggests that volunteer firefighters who recognize their increased risk during the COVID-19 pandemic and who have strong social support may experience post-traumatic growth that can help them adapt and manage during this challenging time. Furthermore, gratitude and the ability to appreciate good things during times of trouble are important components of resilience and can assist with healthy coping [38].

The volunteer firefighters in our study had, on average, volunteered for a long period of time. They expressed pride and enthusiasm for their role in serving the public. These extraordinary individuals routinely confront environmental, biological, and physical dangers. We found that the added risks of COVID-19 infection along with the social turbulence that has characterized the pandemic has placed additional strain on both the professional and personal lives of these individuals. The salience of these findings suggests the important role of leaders who can proactively address mental health challenges, reinforce healthy coping strategies, and encourage resilience among these volunteers.

One unique finding in this study is the impact of the pandemic on deepening the commitment to serve. In the firefighter and EMS communities, significant workforce turnover has led to communities being concerned about staffing of their emergency services. While staffing shortages were a concern prior to the emergence of COVID-19 [39], these have now been exacerbated by the impacts of the pandemic [40–42]. Such staffing shortages can have substantial negative impacts both on the firefighter and EMS workforce, such as longer shifts, and on the public, such as longer wait times after calling 911 [43]. This can be particularly concerning when shortages involve areas where volunteer firefighters and EMS professionals may be the only available first responders [44]. Our finding that members of the volunteer community still found reasons to serve their community in spite of the stress of the pandemic is heartening. Future work can evaluate the impact of the pandemic on volunteerism in other sectors.

Several limitations should be considered when interpreting our study findings. First, our respondents were, on average, slightly older than the typical volunteer firefighter, thus their experiences and concerns may be somewhat different than those of younger volunteers. Second, about one-third of our respondents had a rank of lieutenant or higher, and therefore senior-level volunteers may be somewhat over-represented in our data. Nonetheless, we feel that the perspectives provided by this experienced group are important for understanding the broad impact of the pandemic on the lives and livelihoods of volunteer firefighters. Future work studying a different population of volunteer firefighters may provide additional insight about these issues.

## 5. Conclusions

The impacts of COVID-19 and the ongoing pandemic on the infrastructure of emergency services, particularly in environments that rely on volunteers for these services, have been profound, including in areas such as the safety, mental health, and resilience of first responders. Recognizing the challenges as well as unexpected benefits experienced by these volunteers throughout the pandemic can inform efforts to support these groups and the important work they do for our society.

## Figures and Tables

**Table 1.** Characteristics of interviewees.

Gender	N	%
Female	9	27
Male	24	73
Age (Years)	N	%

20–29	2	6
30–39	3	9
40–49	8	24
50–59	9	27
60–69	10	30
70+	1	3

**Table 2.** Stumbling Blocks for Volunteer Firefighters during the COVID-19 Pandemic.

Role-Related Challenges	Perspectives of Volunteer Firefighters
Fear of COVID exposure and transmission during emergency response	It made me realize just how risky working on an ambulance is. And for my agency we don’t get hazardous duty pay or hazard duty retirement, which is ridiculous, but I can’t really think about it at present, it just made me realize how ridiculous that is.
	I’m really concerned that despite the fact that I have been this vigilant for this long. I’m still going to end up getting it and bring it home to my family and someone I love will still end up dying from it, making this whole pandemic vigilance worthless. That is truly my biggest fear.

Frustration with emergency responders not following public health measures	I found it doubly horrifying that people who say they want to be in service to other people, public, you know, public safety healthcare workers, would not comply.
	A friend of mine does a charitable event for first responders in another state. When they were visiting all the local fire departments about participating in the event, she made a statement that had she worn a mask or asked other people to put on a face covering, that she would have been laughed out of the department. There seems to be this overriding culture of not wanting to follow the public health call and ridiculing anybody that was insisting on it.

Difficulty fostering camaraderie	So typically, we would interact and intermix with them [trainees] more. And I think it created a little bit of a fracture because normally, we would have been interacting with those guys all the time, as they just came into the department. They said they were very isolated to just their own shift. They weren’t interacting with the volunteers, as much. So I think it’s taken a much longer time to develop that interaction and, you know, familiarity.
	It’s not getting to socialize as much. You know, we spend 24 hours in the same building, we’re not really supposed to interact with each other that’s hard to do.

**Table 3.** Personal Stumbling Blocks Related to the COVID-19 Pandemic.

Personal Challenges	Perspectives of Volunteer Firefighters
Fracturing of relationships	Unfortunately, this thing has turned political, which I think is why you have, why people are so passionate on one side or another. My sister-in-law keeps preaching from rooftops all the time she wishes it would stop being political. I wish it wouldn’t have happened, but unfortunately it is. So just trying to get people to sit down and have a calm rational discussion about it has become a real challenge.
	I have learned that some topics are better to not even talk about it because you don’t know if they’re going to explode on you and yell at you knowing you got to work with them. And some people just have a difficult time dealing with the vaccine.

Disruptions to social support system	It really did close people off, but I think more than the adults. But the children are the biggest sufferers because they lost their social relationships, you know. My nephew graduated high school and didn’t get to go to his high school graduation.
	I have two pre-teen boys who missed the better part of an entire school year and or did miss an entire school year really. I’m more concerned about the emotional and mental impacts on our children than I am anything that faces me. When I think about juniors and seniors in high school in our community, who missed out on perhaps the best years of their life because of this, they didn’t get to experience those things.

Chronic stress and negative impacts on mental health	I don’t like people as much anymore because of this information and then repeatedly argumentative about things that they don’t understand. I get it, people inherently fear the unknown, but it’s frustrating. I’m incredibly frustrated every day. Doesn’t look like it’s going away anytime soon. If I could, if I could make the same living, I could do anything but healthcare, I would do that.
	I see kids so tired of hearing about the same stuff over and over and over, doesn’t matter if it’s on the Internet, or TV or radio or whatever, where they’re constantly talking about the good and the bad of the pandemic. After a while you just get tired of hearing about it, so it, does it, does stress you out? Yes. Does it make you more tired? Probably. But is it something you will keep hearing about? Probably.

Feeling frustrated that people are not taking the pandemic seriously	There are definitely a group of people in the broader community, not just in my town but in the broader community to county, that were against the masks. I’m sure that a lot of them embrace anti vax viewpoints, and I don’t think that’s going to stop. You know that they are of that mindset. And it’s frustrating to watch it, it’s pretty scary to watch it.
	Our citizens are refusing to get vaccinated in just numbers that don’t make sense to me, and that’s frustrating.

**Table 4.** Silver Linings of Serving as a Volunteer Firefighter during the COVID-19 Pandemic.

Perceived Benefits	Perspectives of Volunteer Firefighters
Reinforcement of commitment to serve as volunteer firefighters	We try to maintain the fact that we’re doing it for other people. You know we’re all volunteering to serve the community. So, everybody was under the same impression that we can’t stop doing what we do on a daily basis. We need to keep doing it and COVID or not, that’s not an excuse. You know, we’ll do our best to stay away from each other and protect each other, but in the end, it wasn’t for us, it was for them. And that’s kind of how we view everything that we do.
	Oddly enough, we gained more volunteers during the pandemic. Number of individuals actually attending our training nights, our drills, has increased.

Opportunity to improve training and work safety	Flexibility really was something that came out of this where we could have our firefighters do training, which helps our people that were missing training due to work schedules, family emergencies, vacations, and stuff. Now instead of missing the training they were able to do it online at their own leisure. And so, I do believe that is a huge plus that came out of COVID is now the awareness to doing webinars and zoom meetings, you know, facetiming people, online courses and stuff.
	So, I think one of the things that it has done is it has helped us to think about cleaning our equipment, and our facilities, more frequently. I can tell you in the 26 years of being a firefighter, you didn’t go out clean the steering wheel in that fire truck, ever … so, I can tell you that our trucks are getting clean, definitely after every call. So, that’s a huge plus.

**Table 5.** Personal Silver Linings of the COVID-19 Pandemic.

Personal Benefits	Perspectives of Volunteer Firefighters
Developing stronger connections with others	We talked with people about our different things that we’re going through and honestly have been open with our friends about it. And our friends have been really a big help.
	I’ve spent a whole lot more time at home. I spent a whole lot more time with my family.

Having a new outlook on life	I think it’s brought some proposed perspective to what’s really important in life, you know, quality of life, family time, those types of things. Because when I look back at where I was just a year ago or two years ago, I was pretty high strung. Now I’m a lot more relaxed than I ever was just, you know, again, figuring out what the things are in life that are really important in your house, your family, you know, your beliefs, all of those things, and putting that back into perspective.
	I’ve made my choice that the most important thing right now is my children and my grandchildren.

Observing the best of our society	I’m happy to do it, the folks in both of the departments I do it with are just extraordinary. I mean, these people will roll out of bed at 2 in the morning, in the middle of, you know, horrible weather, and go out. They help somebody they might not even know.
	Local grocery stores right off the bat teamed up with the Lion’s Club together for the elderly and they would deliver their groceries. So, a lot of people did their part and you don’t see that and they most certainly were doing the right thing for the elderly. That’s a good thing.

## Data Availability

The data presented in this study are available on request from the corresponding author. The data are not publicly available due to participant privacy concerns.
